# Clinical significance of peripheral blood and tumor tissue lymphocyte subsets in cervical cancer patients

**DOI:** 10.1186/s12885-020-6633-x

**Published:** 2020-03-04

**Authors:** Yutuan Wu, Shuang Ye, Shyamal Goswami, Xuan Pei, Libing Xiang, Xiaoming Zhang, Huijuan Yang

**Affiliations:** 1 Department of Gynecological Oncology, Fudan University Shanghai Cancer Center, Fudan University, Shanghai, China; 2 Department of Oncology, Shanghai Medical College, Fudan University, Shanghai, 200032 China; 3 Unit of Innate Defense and Immune Modulation, Key Laboratory of Molecular Virology and Immunology, Institute Pasteur of Shanghai, Chinese Academy of Sciences, Shanghai, China

**Keywords:** Cervical cancer, Lymphocyte subsets, Tumor immunology, Clinicopathological variables, Prognosis

## Abstract

**Background:**

Alterations in peripheral blood lymphocytes in cervical cancer have been reported, although conflicting views exist. The present study investigated the distributions of lymphocyte subsets in tumor tissue and peripheral blood samples from cervical cancer patients and precancerous lesion patients, and evaluated the correlations of lymphocyte subsets with clinicopathological and prognostic variables.

**Methods:**

A total of 44 patients with stage IB1-IIA2 cervical cancer and 13 precancerous lesion patients were included. Lymphocytes were collected from the tumor tissue and the peripheral blood, and isolated by Lymphoprep density gradient centrifugation. The percentages of lymphocyte subsets were quantified by flow cytometry analysis, and the differences between lymphocyte subsets in the tumor tissue and peripheral blood were compared by SPSS. In addition, the relationships between lymphocyte subsets and clinicopathological and prognostic variables were analyzed.

**Results:**

Our results revealed that the amount of total T lymphocytes, CD8+ T cells, granulocytes, pDCs, CD16+ monocytes and CD56^high^ NK cells were significantly higher in the tumor tissue than in the peripheral blood in the cervical cancer patients, while those of CD4+ T cells, CD4+/CD8+ cell ratio, rdT cells, BDCA1+ mDCs, total monocytes, CD14+ monocytes, NK cells and CD56^low^ NK cells exhibited the opposite trend (*p* < 0.05). The levels of total pDCs and BDCA1+ mDCs in the peripheral blood were significantly lower in the cervical cancer patients than in the precancerous lesion patients, while the proportion of CD16+ monocytes was elevated (*p* < 0.05). In addition, some lymphocyte subsets, especially CD4+ cells and CD8+ cells, and the CD4+/CD8+ cell ratio were closely associated with clinicopathological and prognostic parameters.

**Conclusions:**

These results suggested that distinct alterations in infiltrating lymphocyte subsets occurred in the tumor and were associated with clinicopathological and prognostic parameters. Systemic impairment of the immune system may occur in the antitumor response of cervical cancer patients.

## Background

Cervical cancer is still the leading gynecological malignancy in China, with 98,900 new cases diagnosed in 2015 and over 30,500 deaths [1]. It has been widely accepted that persistent high-risk human papillomavirus (HPV) infection is the major risk factor for cervical cancer [2, 3]. Furthermore, the local immune response is regarded as a key determinant in cervical carcinogenesis after HPV infection [4]. Evidence suggests that cell-mediated immunity may be especially important in HPV-related malignancies, including cervical cancer [5].

Quite a few publications have tried to investigate the immunological status in cervical cancer and/or precancerous lesions [5–15]. Colleagues from France characterized T cells (CD4+, CD8+, CD45 RO+) by immunohistochemistry in high-risk HPV-infected cervical cancers and premalignant lesions [8]. Using flow cytometry, Das et al. assessed peripheral blood lymphocyte subpopulations in cervical cancer patients among Indian women, including only five parameters (CD4+ helper T cells, CD8+ cytotoxic T cells, CD16+ cells, CD19+ cells and CD56+ cells) [5]. Another interesting publication specifically described systemic T cell responses to HPV 16 and HPV 18 proteins [11]. In our previous work (a manuscript published in Chinese), we evaluated CD4^+^CD25^high^CD127^low^ regulatory T cells in the peripheral blood of cervical cancer and precursor lesion patients [16]. We found that regulatory T cells were increased in the patients with cervical intraepithelial neoplasia or cancer [16].

The current study had several objectives. First, this study aimed to describe the immune cell landscape in cervical cancer patients relatively comprehensively, including the landscapes in the peripheral blood, tumor tissue and precancerous lesions. Second, we sought to evaluate possible correlations between lymphocyte subsets and clinicopathological variables. Last, the prognostic implications of immune cell levels were also investigated.

## Methods

### Patient selection and sample collection

We obtained approval from the institutional review board at Fudan University Shanghai Cancer Center (SCCIRB-090371-2). Cervical cancer patients who were scheduled for radical hysterectomy were enrolled in this study between April and November 2013. The included patients were required to fulfill the following criteria: 1) preoperative diagnosis of squamous cell carcinoma or adenocarcinoma of the cervix; 2) International Federation of Gynecology and Obstetrics (FIGO) stage IB1-IIA2 disease, with visible gross tumor; 3) no receipt of preoperative treatment including chemotherapy and radiotherapy; and 4) provision of informed content. Patients with a medical history of autoimmune diseases were excluded. All patients were human immunodeficiency virus (HIV) negative. Clinicopathological information was retrospectively abstracted from patient electronic medical records by a well-trained gynecological oncologist.

Peripheral blood was collected on the day before surgery. Attending surgeons were responsible for choosing and dissecting highly suspicious fresh tumor tissue for further analysis. We also collected peripheral blood samples from 13 patients who received cervical conization for precancerous lesions during the same period.

All the cervical cancer patients at our institution were required to have regular follow-up visits after surgery. Progression-free survival (PFS) was defined as the time interval from the date of primary surgery to the date of disease progression or recurrence.

### Cell isolation from tumor tissue

Freshly resected cervical tissue samples from primary tumors were transferred to the laboratory immediately after surgery and processed within four hours. The fresh tumor tissue was gently minced into 3 to 4 mm cubes using a scalpel, followed by enzymatic digestion in a cocktail of collagenase type IV (1 mg/mL, Gibco, Carlsbad, CA, USA), and DNase I (100 μg/mL, Sigma, USA) in RPMI 1640 medium (HyClone, Utah, USA) and then processed into single-cell suspensions using a gentleMACS Octo Dissociator with Heater (Miltenyi, Bergisch Gladbach, Germany) in the preprogrammed human tumor mode-II at 37 °C for 60 min. Released cells were collected and filtered through 70 μm nylon cell strainers (BD, CA, USA) and processed for density gradient centrifugation to isolate leukocytes.

### Peripheral blood mononuclear cell (PBMC) isolation and staining

Peripheral blood mononuclear cells (PBMCs) were collected by Lymphoprep (Axis-Shield) density gradient centrifugation of venous peripheral blood from patients treated with the anticoagulant K3-ethylenediaminetetraacetic acid (EDTA, BD Cat No. 6457). For whole-blood staining, samples underwent erythrolysis with an ammonium-buffered solution (BD FACS Lysing Solution, 10 min at RT). The cells were resuspended in ice-cold Fluorescence Activated Cell Sorter (FACS) buffer containing 1x phosphate-buffered saline (PBS) (Ca/Mg^++^ free, pH 7.2), 2.5 mM EDTA, 25 mM HEPES and 1% FBS. Samples were assessed for the expression of 18 markers and stained with Zombie Yellow (BioLegend), which was used to discriminate live and dead cells according to the manufacturers’ recommendation, prior to surface staining. For surface staining, filtered single cells were incubated for 30 min on ice with an Fc receptor binding inhibitor (eBioscience) diluted 1/10 in PBS. The cells were then incubated in FACS buffer with combinations of fluorochrome-conjugated or biotinylated antibodies for 15 min in the dark at room temperature. The cells were centrifuged, and the pellets were recovered, fixed in a 1% paraformaldehyde solution and filtered through a 70 μm mesh strainer to remove any small clumps immediately before flow cytometry analysis.

### Flow cytometry analysis

The antibodies and clones used in the study are presented in the Additional file 1. Samples were acquired using a BD LSRFortessa flow cytometer (BD Biosciences) equipped with five lasers. Before running samples, the alignment of the instrument was checked daily using fluorescent microbeads; also, the accuracy of the volumetric apparatus was addressed by using commercial fluorescent beads having predetermined acceptable ranges for validation. Compensation was performed according to the cytometer manufacturer’s instructions, allowing the use of an off-line procedure by applying automated electronic algorithms and preset templates; by using biparametric logarithmic dot plots, gate-specific tubes and single-tube data analysis; and by optimizing the FSC threshold and fluorochrome voltage as set-up parameters. All the data were analyzed with FlowJo software 9.7.3 (TreeStar, Inc. Ashland, OR).

### Statistical analyses

Statistical Package for Social Science (SPSS) statistical software (Version 20.0, SPSS, Inc., Chicago, IL, USA) was used for analyses. Continuous data are presented as the median (range), and categorical data are presented as proportions. Parametric Student’s t tests were employed to evaluate continuous variables. The associations between different variables were evaluated using univariate and multivariate logistic regression analysis, and hazard ratios (HRs) with 95% confidence intervals (CIs) were calculated. All of the *P* values reported are two sided, and a value of *P* < 0.05 was considered statistically significant.

## Results

### Demographics

**Fig. 1:**
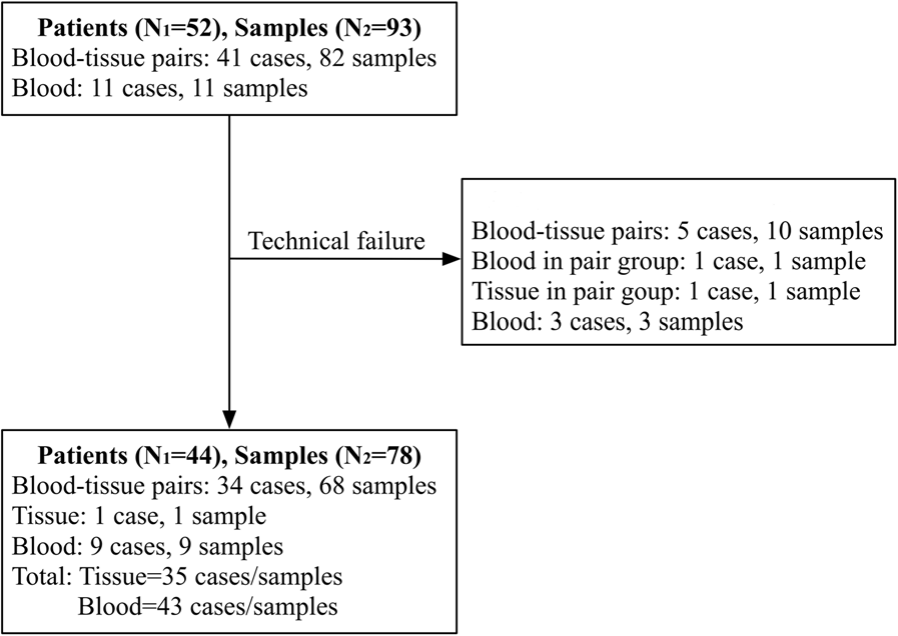
Schematic of patients included in the present study

Figure 1 shows the schematic of patient enrollment in the study. A total of 44 patients were ultimately included, and 78 samples were available for analysis. Among the patients, 34 had paired blood-tissue samples. Clinical and pathological information for the entire cohort is presented in Table 1. The majority of the patients had a squamous histology (*n* = 40, 90.9%). Deep stromal invasion (≥ 1/2 depth), lymph node metastasis and positive lymphovascular invasion occurred in 70.5, 40.9 and 47.7% of the patients, respectively. With a median follow-up time of 41 months, 36 patients (81.8%) were alive with no evidence of disease, while 8 patients (18.2%) had experienced disease recurrence.

**Table 1:** Patient characteristics (*n* = 44)

Median age (range), years	47 (23–70)
FIGO stage	
IB1 (%)	15 (34.1%)
IB2 (%)	8 (18.2%)
IIA1 (%)	11 (25.0%)
IIA2 (%)	10 (22.7%)
Histology	
Squamous carcinoma (%)	40 (90.9%)
Adenocarcinoma (%)	4 (9.1%)
Stromal invasion ≥1/2 depth (%)	31 (70.5%)
Lymph node metastasis (%)	18 (40.9%)
Positive lymphvascular invasion (%)	21 (47.7%)
Median follow up (range), months	41 (2–48)
Disease status at last follow-up	
Alive, no evidence of disease (%)	36 (81.8%)
Alive, with disease (%)	5 (11.4%)
Dead with disease (%)	3 (6.8%)

Abbreviations: *FIGO* International Federation of Gynecology and Obstetrics

### Lymphocyte subpopulations in the peripheral blood versus tumor tissue

We collected paired blood-tissue samples from 34 patients, and compared different lymphocyte subsets in the tumor tissue versus the peripheral blood. The distributions of the lymphocyte subsets in the peripheral blood compared with those in the tumor tissue are presented in Table 2.

**Table 2:** Lymphocyte subsets in peripheral blood versus tumor tissue (*n* = 34)

	Blood	Tissue	*P* value*
Gran %CD45	3.24 ± 5.72	10.55 ± 12.71	0.002
T %MNC	55.59 ± 12.03	68.58 ± 17.81	< 0.001
CD4%T	54.04 ± 13.18	45.85 ± 14.49	0.013
CD8%T	32.37 ± 11.22	44.92 ± 13.98	< 0.001
CD4:CD8	1.94 ± 0.94	1.22 ± 0.74	0.001
rdT %T	7.53 ± 4.57	2.68 ± 1.77	0.001
B % MNC	5.93 ± 2.92	5.00 ± 3.44	0.302^#^
pDC % MNC	0.24 ± 0.13	0.67 ± 0.79	0.003
CD11c + % pDC	8.13 ± 5.08	5.90 ± 4.74	0.109^#^
Baso % MNC	0.83 ± 0.50	0.09 ± 0.16	< 0.001
BDCA1+ mDC % MNC	0.37 ± 0.17	0.11 ± 0.14	< 0.001
BDCA3+ mDC % MNC	0.02 ± 0.04	0.02 ± 0.06	1.000^#^
Mono % MNC	16.69 ± 9.38	8.66 ± 8.29	< 0.001
CD14+ Mo % MNC	14.76 ± 7.03	2.28 ± 2.34	< 0.001
CD16+ Mo % MNC	3.27 ± 2.25	5.85 ± 5.75	0.036
CD16+ %Mono	18.45 ± 7.79	71.62 ± 13.20	< 0.001
NK %MNC	17.27 ± 9.83	5.88 ± 12.63	< 0.001
CD56hi NK % MNC	0.33 ± 0.16	4.38 ± 11.71	0.079^#^
CD56lo NK % MNC	17.66 ± 9.97	1.24 ± 1.94	< 0.001
CD56hi %NK	2.42 ± 1.81	65.31 ± 23.69	< 0.001
MDSC % CD33hi	1.85 ± 3.50	2.39 ± 3.83	0.592^#^
DR- %14 + APC	0.41 ± 1.50	18.13 ± 18.68	< 0.001
DR- %16 + APC	0.67 ± 1.08	1.64 ± 2.96	0.059^#^

*Paired t test

^#^*P* values with no statistical significance were denoted

Abbreviations: *Gran* Granulocyte, *MNC* Mononuclear Cell, *DC* Dendritic Cell, *pDC* Plasmacytoid Dendritic Cell, *mDC* Myeloid Dendritic Cell, *NK* Natural Killer, *hi* high, *lo* low, *MDSC* Myeloid-Derived Suppressor Cell, *APC* Antigen Presenting Cell, *Baso* Basophilic granulocyte, *Mono* Monocyte;

#### Distributions of T lymphocytes, granulocytes and B lymphocytes

The amount of total T lymphocytes and granulocytes were significantly higher in the tumor tissue than in the peripheral blood(*p* < 0.05). However, a significant decrease in the T helper lymphocyte (CD4+ cell) population in the tumor tissue was observed compared to that in the peripheral blood (*p* < 0.05), while the level of T suppressor lymphocytes (CD8+ cells) was found to be significantly increased in the tumor tissue compared to the peripheral blood (*p* < 0.05). The CD4+/CD8+ cell ratio was significantly lower in the tumor tissue than in the peripheral blood (*p* < 0.05). Furthermore, the proportion of rdT cells, which play an important role in the function of innate immunity, was significantly lower in the tumor tissue than in the peripheral blood (*p* < 0.05). All of these results indicated that antitumor immunity was suppressed in the tumor tissue. However, no significant difference was observed for B lymphocytes when comparing the tumor tissue with the peripheral blood (*p* > 0.05).

#### Distributions of dendritic cells and basophilic granulocytes

Dendritic cells (DCs) shape both innate and adaptive immune responses, including antitumor immune responses. There are two main types of DCs that are distinguished on the basis of different sources of differentiation, plasmacytoid dendritic cells (pDCs) and myeloid dendritic cells (mDCs). The proportion of pDCs, which are considered to induce more T regulatory cells (Tregs) and to promote immune escape by tumor cells, was significantly higher in the tumor tissue than in the peripheral blood (*p* < 0.05). However, CD11c + pDCs, a major kind of pDCs, showed no significant difference between the tumor tissue and peripheral blood. Recently, two subsets of naturally occurring human blood-derived mDCs, BDCA1+/CD1c + mDCs and BDCA3+/CD141+ mDCs, have been described. Our results indicated that the level of BDCA1+ mDCs was significantly lower in the tumor tissue than in the peripheral blood (*p* < 0.05). However, there was no significant difference in BDCA3+ mDCs between the tumor tissue and peripheral blood. In addition, the proportion of basophilic (Baso) granulocytes was significantly lower in the tumor tissue than in the peripheral blood (*p* < 0.05).

#### Distributions of monocytes, NK cells and myeloid-derived suppressor cells

Total monocyte and CD14+ monocyte frequencies were significantly decreased in the tumor tissue compared to the peripheral blood (*p* < 0.05). In contrast, the CD16+ monocyte frequency was significantly higher in the tumor tissue than in the peripheral blood (*p* < 0.05). Moreover, the subsets of natural killer cells (NK cells), which are believed to have the function of immune surveillance, were significantly decreased in the tumor tissue compared to the peripheral blood (*p* < 0.05). The CD56^high^ NK cell proportion was significantly higher in the tumor tissue than in the peripheral blood (*p* < 0.05), but the CD56^low^ NK cell proportion exhibited the opposite trend (*p* < 0.05). Furthermore, myeloid-derived suppressor cells (MDSCs) showed no significant difference between the tumor tissue and peripheral blood.

### Lymphocyte subpopulations in the peripheral blood: cervical cancer versus precancerous lesions

We detected the levels of different lymphocyte subpopulations in peripheral blood samples from cervical cancer patients and precancerous lesion patients. The results showed that the percentages of total pDCs and BDCA1+ mDCs were significantly lower in the cervical cancer patients than in the precancerous lesion patients; meanwhile, the CD16+ monocyte and CD56^low^ NK cell proportions were higher in the cervical cancer patients than in the precancerous lesion patients, and the differences were significant between the cervical cancer patients and precancerous lesion patients (*p* < 0.05) (Table 3). However, other lymphocyte subpopulations were not significantly different between the cervical cancer patients and precancerous lesion patients (*p* > 0.05) (Table 3).

**Table 3:** Lymphocyte subsets in peripheral blood: cervical cancer versus precancerous lesion

	Cancer (*n* = 43)	Precancerous lesion (*n* = 13)	*P* value*
Gran %CD45	3.05 ± 5.29	4.17 ± 6.17	0.522
T %MNC	57.41 ± 12.79	59.58 ± 9.54	0.575
rdT %T	7.30 ± 4.45	4.83 ± 2.22	0.067
B %MNC	6.05 ± 3.11	6.48 ± 2.48	0.644
pDC % MNC	0.25 ± 0.13	0.35 ± 0.16	0.022^#^
CD11c + % pDC	7.62 ± 5.03	6.18 ± 1.97	0.386
Baso % MNC	0.98 ± 0.71	1.19 ± 0.60	0.334
BDCA1+ mDC % MNC	0.38 ± 0.18	0.65 ± 0.23	< 0.001^#^
BDCA3+ mDC % MNC	0.17 ± 0.04	0.03 ± 0.05	0.374
Mono %MNC	15.56 ± 9.24	17.62 ± 8.01	0.473
CD14+ Mo % MNC	14.63 ± 6.98	16.98 ± 7.00	0.359
CD16+ Mo % MNC	3.07 ± 2.19	2.52 ± 1.04	0.448
CD16+ %Mono	15.58 ± 7.86	13.36 ± 3.29	0.020^#^
NK %MNC	16.11 ± 9.34	10.90 ± 4.91	0.060
CD56hi NK % MNC	0.33 ± 0.15	0.29 ± 0.19	0.508
CD56lo NK % MNC	17.26 ± 9.62	10.36 ± 5.33	0.037^#^
CD56hi %NK	2.54 ± 2.05	3.93 ± 3.03	0.106
MDSC % CD33hi	1.85 ± 3.32	3.42 ± 7.74	0.547
DR- %14 + APC	0.51 ± 1.55	0.38 ± 0.95	0.800
DR- %16 + APC	0.88 ± 1.60	0.44 ± 0.75	0.404

*Independent t test

^#^*P* values with statistical significance were denoted

### Associations of lymphocyte subsets with clinicopathological parameters

We correlated the distributions of lymphocyte subsets with clinicopathological parameters, such as age, FIGO stage, histology, tumor size, stromal invasion status, lymph node (LN) metastasis status and lymphovascular space invasion (LVSI) status.

#### Age, tumor size and LVSI status are closely related to the distributions of lymphocytes in the peripheral blood

In the analysis of the peripheral blood, the results showed that the CD16+ monocyte proportion was significantly lower in the age ≤ 47 group than in the age > 47 group (*p* < 0.05) (Table 4). Additionally, total T cell and CD56^high^ NK cell proportions were significantly lower in the tumor size ≤4 cm group than in the tumor size > 4 cm group, while the BDCA3+ mDC, total NK cell and CD56^low^ NK cell proportions exhibited the opposite trend (*p* < 0.05) (Table 4). The CD11c + pDC proportion was significantly lower in the LVSI-positive patients than in the LVSI-negative patients (*p* < 0.05) (Table 4). In addition, there were no other significant differences between clinicopathological parameters and lymphocyte subsets in the peripheral blood.

**Table 4:** Associations between lymphocyte subsets and clinicopathological parameters

	Age (years)			FIGO stage			Histology			Tumor size			Stromal invasion			LN metastasis			LVSI		
	≤47	> 47	*P*	I	II	*P*	SCC	ADC	*P*	≤4 cm	> 4 cm	*P*	≤1/2	> 1/2	*P*	No	Yes	*P*	No	Yes	*P*
Blood (n = 43)																					
Gran %CD45	3.47 ± 6.06	2.45 ± 4.08	0.537	2.17 ± 3.92	4.06 ± 6.49	0.266	2.68 ± 4.95	5.82 ± 7.50	0.216	2.43 ± 3.39	3.83 ± 7.03	0.431	2.34 ± 4.88	3.35 ± 5.51	0.57	1.76 ± 2.76	4.84 ± 7.25	0.101	2.01 ± 3.94	4.13 ± 6.33	0.198
T %MNC	57.57 ± 12.26	57.19 ± 13.85	0.925	56.69 ± 12.69	58.25 ± 13.19	0.694	57.70 ± 13.39	55.22 ± 7.18	0.688	53.35 ± 10.22	62.55 ± 14.08	0.017	56.56 ± 14.17	57.78 ± 12.38	0.777	56.88 ± 13.82	58.16 ± 11.55	0.751	56.44 ± 13.59	58.44 ± 12.14	0.614
CD4%T	57.49 ± 9.65	53.42 ± 15.93	0.304	57.17 ± 11.74	54.19 ± 13.76	0.447	55.41 ± 13.00	59.43 ± 9.07	0.552	58.25 ± 10.16	52.67 ± 14.94	0.153	59.35 ± 10.65	54.24 ± 13.30	0.229	58.17 ± 10.08	52.47 ± 15.23	0.147	56.98 ± 10.72	54.54 ± 14.57	0.534
CD8%T	31.18 ± 7.98	31.94 ± 13.81	0.821	29.73 ± 9.48	33.54 ± 11.79	0.248	31.95 ± 10.92	27.10 ± 7.15	0.392	29.64 ± 8.32	33.86 ± 12.89	0.201	30.75 ± 9.19	31.83 ± 11.37	0.764	29.36 ± 7.71	34.48 ± 13.44	0.121	30.70 ± 8.18	32.34 ± 12.92	0.619
CD4:CD8	2.06 ± 0.92	2.03 ± 1.04	0.936	2.22 ± 1.05	1.85 ± 0.83	0.204	2.02 ± 0.97	2.35 ± 0.87	0.513	2.20 ± 0.94	1.85 ± 0.97	0.242	2.22 ± 1.09	1.97 ± 0.91	0.44	2.19 ± 0.89	1.85 ± 1.04	0.254	2.07 ± 0.92	2.02 ± 1.02	0.873
rdT %T	7.2 ± 4.69	7.45 ± 4.32	0.91	6.10 ± 3.70	8.90 ± 5.06	0.16	7.42 ± 4.72	6.60 ± 2.75	0.775	7.23 ± 4.35	7.46 ± 4.99	0.915	5.07 ± 3.49	8.42 ± 4.55	0.105	7.43 ± 4.07	7.00 ± 5.71	0.848	6.67 ± 4.33	8.57 ± 4.73	0.369
B %MNC	6.05 ± 3.07	6.03 ± 3.25	0.983	6.13 ± 3.50	5.96 ± 2.71	0.86	5.99 ± 3.14	6.44 ± 3.18	0.766	6.30 ± 3.24	5.71 ± 2.99	0.546	6.40 ± 3.10	5.90 ± 3.16	0.646	6.61 ± 3.48	5.22 ± 2.32	0.157	7.03 ± 3.42	5.06 ± 2.47	0.039
pDC % MNC	0.26 ± 0.13	0.23 ± 0.11	0.415	0.26 ± 0.14	0.24 ± 0.11	0.583	0.25 ± 0.13	0.24 ± 0.05	0.904	0.23 ± 0.11	0.27 ± 0.14	0.212	0.25 ± 0.16	0.24 ± 0.11	0.805	0.24 ± 0.12	0.26 ± 0.14	0.526	0.22 ± 0.12	0.28 ± 0.13	0.133
CD11c + % pDC	7.71 ± 6.14	7.49 ± 2.98	0.909	8.09 ± 6.31	7.01 ± 2.76	0.553	7.40 ± 4.94	9.13 ± 6.24	0.531	6.74 ± 3.28	8.91 ± 6.80	0.237	9.31 ± 8.32	6.96 ± 2.98	0.429	7.83 ± 3.63	7.27 ± 6.95	0.765	9.24 ± 6.28	5.78 ± 2.05	0.044
Baso % MNC	1.09 ± 0.80	0.83 ± 0.54	0.248	1.07 ± 073	0.89 ± 0.69	0.41	0.92 ± 0.65	1.60 ± 1.00	0.065	1.02 ± 0.65	0.93 ± 0.78	0.686	1.08 ± 0.59	0.94 ± 0.76	0.534	1.00 ± 0.70	0.95 ± 0.73	0.808	1.03 ± 0.58	0.93 ± 0.83	0.637
BDCA1+ mDC % MNC	0.39 ± 0.18	0.35 ± 0.19	0.547	0.34 ± 0.23	0.42 ± 0.10	0.21	0.36 ± 0.18	0.57 ± 0.12	0.062	0.38 ± 0.14	0.37 ± 0.24	0.826	0.29 ± 0.20	0.41 ± 0.17	0.087	0.37 ± 0.22	0.39 ± 0.12	0.717	0.32 ± 0.21	0.44 ± 0.14	0.074
BDCA3+ mDC % MNC	0.02 ± 0.04	0.02 ± 0.04	0.875	0.02 ± 0.04	0.01 ± 0.03	0.237	0.01 ± 0.04	0.05 ± 0.07	0.203	0.03 ± 0.05	0	0.02	0.03 ± 0.05	0.01 ± 0.04	0.477	0.02 ± 0.04	0.02 ± 0.04	0.871	0.02 ± 0.04	0.01 ± 0.04	0.754
Mono %MNC	17.19 ± 9.54	13.30 ± 8.56	0.177	15.88 ± 9.83	15.19 ± 8.75	0.81	15.48 ± 9.60	16.14 ± 6.60	0.884	16.14 ± 7.88	14.83 ± 10.91	0.651	15.80 ± 11.75	15.46 ± 8.16	0.925	13.82 ± 8.77	17.97 ± 9.59	0.149	15.61 ± 10.16	15.50 ± 8.43	0.97
CD14+ Mo % MNC	16.31 ± 7.38	12.18 ± 5.76	0.101	13.64 ± 7.69	15.91 ± 5.99	0.371	14.53 ± 7.17	15.38 ± 6.35	0.824	14.37 ± 6.06	15.02 ± 8.41	0.802	13.21 ± 10.31	15.19 ± 5.38	0.597	13.01 ± 6.89	17.33 ± 6.53	0.09	13.81 ± 8.44	15.56 ± 4.97	0.476
CD16+ Mo % MNC	2.68 ± 1.65	3.65 ± 2.77	0.274	2.82 ± 2.47	3.39 ± 1.79	0.473	3.14 ± 2.32	2.60 ± 0.89	0.652	3.57 ± 2.49	2.35 ± 1.44	0.09	2.44 ± 2.23	3.32 ± 2.17	0.332	2.78 ± 1.95	3.57 ± 2.54	0.329	2.54 ± 1.80	3.68 ± 2.47	0.142
CD16+ %Mono	14.68 ± 6.43	21.81 ± 8.06	0.009	17.03 ± 8.41	18.29 ± 7.33	0.661	17.75 ± 7.85	16.38 ± 8.98	0.749	19.47 ± 7.83	14.81 ± 7.32	0.1	16.34 ± 7.16	18.06 ± 8.22	0.567	17.77 ± 7.2	17.27 ± 8.72	0.865	16.65 ± 8.29	18.63 ± 7.48	0.485
NK %MNC	14.08 ± 7.89	19.08 ± 10.70	0.089	16.16 ± 10.07	16.05 ± 8.66	0.97	15.92 ± 9.79	17.88 ± 2.54	0.696	19.52 ± 9.59	11.56 ± 6.89	0.005	16.06 ± 13.23	16.13 ± 7.26	0.983	17.82 ± 11.08	13.58 ± 5.30	0.106	15.93 ± 10.99	16.30 ± 7.40	0.9
CD56hi NK % MNC	0.33 ± 0.15	0.33 ± 0.15	0.935	0.32 ± 0.16	0.34 ± 0.13	0.804	0.33 ± 0.16	0.35 ± 0.06	0.559	0.31 ± 0.15	0.36 ± 0.14	0.3	0.34 ± 0.19	0.32 ± 0.13	0.704	0.35 ± 0.17	0.29 ± 0.11	0.241	0.34 ± 0.18	0.31 ± 0.11	0.596
CD56lo NK % MNC	15.29 ± 8.37	20.13 ± 10.91	0.166	17.23 ± 10.95	17.29 ± 8.01	0.986	17.22 ± 10.28	17.53 ± 2.59	0.954	20.55 ± 9.72	12.45 ± 7.43	0.017	18.57 ± 15.14	16.75 ± 6.79	0.736	18.58 ± 11.44	15.06 ± 5.16	0.244	16.66 ± 11.42	17.94 ± 7.42	0.714
CD56hi %NK	2.73 ± 1.94	2.28 ± 2.26	0.553	2.83 ± 2.56	2.18 ± 1.13	0.345	2.63 ± 2.18	1.98 ± 0.61	0.563	1.72 ± 1.06	3.75 ± 2.57	0.017	2.97 ± 2.53	2.38 ± 1.88	0.476	2.77 ± 2.43	2.18 ± 1.23	0.441	3.06 ± 2.56	1.96 ± 1.09	0.121
MDSC % CD33hi	1.45 ± 2.45	2.44 ± 4.33	0.416	1.49 ± 2.70	2.31 ± 4.04	0.501	2.00 ± 3.52	0.83 ± 0.93	0.518	1.46 ± 2.43	2.42 ± 4.36	0.434	0.70 ± 0.57	2.30 ± 3.83	0.226	0.75 ± 0.68	3.68 ± 4.94	0.065	0.67 ± 0.63	3.19 ± 4.50	0.05
DR- %14 + APC	0.44 ± 1.04	0.62 ± 2.13	0.761	0.87 ± 2.01	0.06 ± 0.09	0.107	0.58 ± 1.65	0.08 ± 0.15	0.554	0.08 ± 0.15	1.15 ± 2.33	0.125	0.29 ± 0.79	0.60 ± 1.77	0.617	0.65 ± 1.89	0.28 ± 0.69	0.525	0.66 ± 1.90	0.35 ± 1.05	0.578
DR- %16 + APC	1.13 ± 1.94	0.53 ± 0.88	0.309	1.38 ± 1.95	0.24 ± 0.61	0.029	0.99 ± 1.69	0.13 ± 0.13	0.319	0.49 ± 0.75	1.46 ± 2.28	0.16	0.60 ± 0.81	1.00 ± 1.83	0.539	1.08 ± 1.90	0.56 ± 0.92	0.381	0.94 ± 1.28	0.82 ± 1.95	0.835
Tissue (*n* = 35)																					
Gran %CD45	11.18 ± 15.73	9.1 ± 7.95	0.643	8.48 ± 10.29	11.93 ± 14.63	0.428	10.71 ± 12.97	5.40 ± 8.50	0.495	8.54 ± 14.45	12.28 ± 10.19	0.391	10.46 ± 16.45	10.18 ± 11.44	0.956	11.38 ± 13.34	8.56 ± 9.84	0.526	13.16 ± 15.69	6.79 ± 6.58	0.12
T %MNC	63.97 ± 21.84	75.21 ± 8.67	0.05	73.72 ± 11.79	64.76 ± 21.53	0.139	68.14 ± 18.30	79.50 ± 6.17	0.298	69.46 ± 20.43	68.70 ± 14.82	0.903	67.14 ± 23.32	69.79 ± 16.01	0.707	71.27 ± 17.29	65.87 ± 18.78	0.388	71.22 ± 15.49	66.61 ± 20.51	0.455
CD4%T	43.98 ± 16.01	47.32 ± 12.53	0.504	48.31 ± 14.14	42.86 ± 14.57	0.27	45.13 ± 14.79	49.57 ± 10.77	0.617	49.15 ± 13.38	41.19 ± 14.82	0.105	50.23 ± 15.25	43.87 ± 14.05	0.26	50.45 ± 11.76	38.10 ± 15.25	0.011	39.88 ± 13.92	52.19 ± 12.29	0.01
CD8%T	47.34 ± 15.92	43.21 ± 11.74	0.397	42.68 ± 12.47	48.06 ± 15.41	0.266	45.68 ± 14.36	42.93 ± 13.46	0.752	41.10 ± 13.04	50.61 ± 13.98	0.045	38.96 ± 12.74	47.70 ± 14.10	0.111	40.08 ± 11.89	53.50 ± 13.69	0.004	50.61 ± 13.71	39.33 ± 12.36	0.016
CD4:CD8	1.17 ± 0.82	1.24 ± 0.66	0.759	1.32 ± 0.71	1.09 ± 0.77	0.374	1.19 ± 0.75	1.31 ± 0.73	0.802	1.41 ± 0.81	0.96 ± 0.58	0.07	1.55 ± 0.96	1.08 ± 0.62	0.099	1.44 ± 0.73	0.85 ± 0.62	0.019	0.92 ± 0.55	1.54 ± 0.80	0.01
rdT %T	3.43 ± 1.84	1.53 ± 1.05	0.014	2.55 ± 1.73	2.60 ± 1.94	0.952	2.42 ± 1.54	3.95 ± 4.03	0.687	2.57 ± 1.94	2.59 ± 1.61	0.985	3.44 ± 2.07	2.11 ± 1.49	0.112	2.61 ± 1.93	2.43 ± 1.21	0.857	2.75 ± 1.98	2.04 ± 0.94	0.454
B %MNC	4.42 ± 3.37	5.78 ± 3.41	0.252	4.67 ± 3.50	5.41 ± 3.38	0.534	5.10 ± 3.32	4.70 ± 5.07	0.851	4.32 ± 2.90	6.00 ± 3.85	0.157	4.04 ± 2.67	5.38 ± 3.59	0.339	4.68 ± 3.00	5.68 ± 4.04	0.41	4.54 ± 2.11	5.65 ± 4.45	0.372
pDC % MNC	0.66 ± 0.68	0.64 ± 0.92	0.94	0.71 ± 0.85	0.59 ± 0.74	0.65	0.65 ± 0.78	0.67 ± 0.98	0.967	0.36 ± 0.32	0.99 ± 1.02	0.028	0.49 ± 0.49	0.70 ± 0.87	0.487	0.67 ± 0.95	0.61 ± 0.46	0.837	0.62 ± 0.69	0.69 ± 0.90	0.792
CD11c + % pDC	5.39 ± 5.60	6.21 ± 3.66	0.647	5.76 ± 3.53	5.81 ± 5.84	0.979	5.64 ± 4.35	7.70 ± 10.89	0.834	5.57 ± 5.03	6.13 ± 4.31	0.764	4.21 ± 4.06	6.38 ± 4.87	0.274	4.77 ± 3.91	7.70 ± 5.64	0.112	4.97 ± 3.57	6.93 ± 5.92	0.321
Baso % MNC	0.11 ± 0.16	0.06 ± 0.16	0.328	0.12 ± 0.20	0.05 ± 0.10	0.192	0.08 ± 0.17	0.13 ± 0.12	0.6	0.06 ± 0.13	0.11 ± 0.19	0.375	0.11 ± 0.18	0.08 ± 0.16	0.591	0.10 ± 0.19	0.07 ± 0.11	0.675	0.08 ± 0.16	0.09 ± 0.17	0.953
BDCA1+ mDC % MNC	0.11 ± 0.07	0.11 ± 0.18	0.882	0.11 ± 0.08	0.11 ± 0.17	0.926	0.11 ± 0.14	0.10 ± 0.00	0.913	0.11 ± 0.18	0.11 ± 0.06	0.904	0.10 ± 0.08	0.11 ± 0.15	0.82	0.15 ± 0.17	0.06 ± 0.05	0.074	0.07 ± 0.07	0.17 ± 0.18	0.057
BDCA3+ mDC % MNC	0.03 ± 0.08	0	0.136	0.01 ± 0.03	0.03 ± 0.08	0.451	0.02 ± 0.06	0	0.682	0.03 ± 0.08	0.01 ± 0.03	0.392	0.04 ± 0.11	0.01 ± 0.03	0.464	0.03 ± 0.08	0.01 ± 0.03	0.392	0.01 ± 0.02	0.03 ± 0.09	0.317
Mono %MNC	10.22 ± 9.84	6.28 ± 5.50	0.164	9.48 ± 9.17	7.41 ± 7.47	0.468	8.78 ± 8.52	4.50 ± 4.04	0.4	5.48 ± 4.78	11.90 ± 10.22	0.032	8.90 ± 11.80	8.25 ± 6.98	0.843	7.42 ± 8.05	9.91 ± 8.71	0.393	10.33 ± 10.36	6.15 ± 4.09	0.119
CD14+ Mo % MNC	2.62 ± 2.76	1.76 ± 1.77	0.329	2.43 ± 2.72	1.96 ± 1.92	0.593	2.28 ± 2.39	1.15 ± 1.20	0.519	1.34 ± 1.31	3.61 ± 2.98	0.034	2.06 ± 2.86	2.26 ± 2.18	0.845	2.35 ± 2.38	1.93 ± 2.36	0.656	2.79 ± 2.78	1.37 ± 1.18	0.071
CD16+ Mo % MNC	6.19 ± 7.02	5.09 ± 4.16	0.615	6.15 ± 5.32	5.12 ± 6.33	0.638	5.78 ± 5.88	4.00 ± 4.38	0.681	3.49 ± 2.98	9.19 ± 7.43	0.032	4.06 ± 6.19	6.26 ± 5.61	0.366	4.88 ± 6.09	7.13 ± 4.99	0.325	5.91 ± 7.01	5.29 ± 3.51	0.781
CD16+ %Mono	68.75 ± 13.39	75.44 ± 12.31	0.174	74.09 ± 13.74	69.72 ± 12.48	0.379	71.65 ± 13.52	76.50 ± 2.55	0.622	72.47 ± 13.28	71.19 ± 13.39	0.804	68.29 ± 14.51	73.39 ± 12.61	0.358	67.45 ± 13.04	80.59 ± 8.24	0.008	68.74 ± 13.19	76.58 ± 12.01	0.113
NK %MNC	7.45 ± 16.28	3.58 ± 4.04	0.376	2.39 ± 1.63	9.10 ± 17.14	0.127	6.00 ± 12.81	1.60 ± 0.28	0.635	7.69 ± 16.47	3.28 ± 2.40	0.313	1.83 ± 0.92	7.15 ± 14.33	0.279	3.86 ± 4.56	8.78 ± 19.40	0.269	3.92 ± 4.75	8.06 ± 18.08	0.343
CD56hi NK % MNC	5.69 ± 15.71	2.75 ± 3.90	0.502	1.56 ± 1.51	7.18 ± 16.30	0.221	4.54 ± 11.91	0.65 ± 0.21	0.653	5.95 ± 14.48	1.53 ± 1.33	0.325	1.34 ± 0.69	5.39 ± 13.45	0.407	2.33 ± 3.33	7.97 ± 19/17	0.379	2.28 ± 3.49	7.10 ± 17.47	0.274
CD56lo NK % MNC	1.42 ± 2.55	0.97 ± .88	0.538	0.87 ± 0.86	1.56 ± 2.61	0.345	1.23 ± 1.98	0.90 ± 0.14	0.821	1.23 ± 2.34	1.15 ± 0.96	0.917	0.44 ± 0.41	1.50 ± 2.18	0.188	0.98 ± 0.85	1.63 ± 3.10	0.393	0.98 ± 0.89	1.52 ± 2.82	0.469
CD56hi %NK	69.20 ± 19.96	63.24 ± 27.93	0.512	59.81 ± 26.37	73.30 ± 19.46	0.128	68.18 ± 23.71	41.25 ± 2.47	0.126	72.92 ± 22.72	55.53 ± 22.66	0.055	77.68 ± 16.96	62.00 ± 25.04	0.068	65.42 ± 24.16	68.03 ± 24.56	0.785	64.04 ± 24.68	69.56 ± 23.29	0.549
MDSC % CD33hi	2.58 ± 3.27	2.22 ± 4.34	0.803	1.16 ± 1.92	3.74 ± 4.77	0.076	2.55 ± 3.86	0.50 ± 0.71	0.467	2.71 ± 4.15	1.92 ± 3.15	0.594	1.99 ± 2.98	2.57 ± 4.07	0.718	2.94 ± 4.48	1.39 ± 1.47	0.299	3.22 ± 4.66	1.26 ± 1.39	0.118
DR- %14 + APC	12.53 ± 18.65	23.71 ± 16.82	0.102	19.69 ± 20.97	16.04 ± 15.69	0.602	19.25 ± 18.36	/	/	14.93 ± 15.86	22.83 ± 21.80	0.269	15.24 ± 18.16	18.95 ± 18.80	0.636	21.95 ± 19.33	10.27 ± 14.27	0.105	23.35 ± 19.78	10.23 ± 13.43	0.057
DR- %16 + APC	1.64 ± 2.56	1.54 ± 3.35	0.931	2.06 ± 3.67	1.09 ± 1.83	0.382	1.59 ± 3.00	1.65 ± 2.19	0.978	1.56 ± 3.05	1.65 ± 2.83	0.931	2.81 ± 4.28	1.13 ± 2.17	0.169	2.37 ± 3.37	0.11 ± 0.16	0.009	2.42 ± 3.54	0.42 ± 0.95	0.038

#### Age, tumor size, LN metastasis status and LVSI status are closely related to the distributions of lymphocytes in tumor tissue

In tumor tissue, the rdT cell proportion was significantly higher in the age ≤ 47 group than in the age > 47 group (*p* < 0.05) (Table 4). The levels of CD8+ cells, pDCs, total monocytes, CD14+ monocytes and CD 16+ monocytes were significantly higher in the tumor size > 4 cm group than in the tumor size ≤4 cm group (*p* < 0.05) (Table 4). Our results indicated that tumor size was closely related to the distributions of some lymphocyte subsets in both the peripheral blood and the tumor tissue. LN metastasis was correlated with significant reductions in the level of CD4+ cells and the CD4+/CD8+ cell ratio but also with increased levels of CD8+ cells and CD16+ monocytes (*p* < 0.05) (Table 4). Contradictorily, LVSI was correlated with a significantly reduced level of CD8+ cells but also with an increased level of CD4+ cells and an elevated CD4+/CD8+ cell ratio (*p* < 0.05) (Table 4). In addition, no statistically significant differences were found in other lymphocyte subsets or clinicopathological variables.

### Prognostic implications of lymphocyte subpopulations

#### The CD4+/CD8+ cell ratio differed significantly in both the peripheral blood and the tumor tissue

We analyzed the correlations of 43 patients’ lymphocyte subsets in the peripheral blood with basic characteristics, and the results revealed that most of the basic characteristics, such as age (≤47 years vs. > 47 years), stage (FIGO I vs. ≥FIGO II), tumor size (≤4 cm vs. > 4 cm) and stromal invasion (≤1/2 vs. > 1/2), did not exhibit significant difference, but histology (SCC vs. ADC), LN metastasis status (negative vs. positive) and LVSI status (negative vs. positive) were found to be statistically significant (*p* < 0.05) (Table 5). When comparing the low level with the high level of a lymphocyte subset (the median value of the lymphocyte subset was used as the cut-off value), we found that most lymphocyte subsets did not exhibit significant differences (*p* > 0.05); however, the difference in the CD4+/CD8+ cell ratio was significant (*p* < 0.05) (Table 5).

**Table 5:** Prognostic implication of lymphocyte subpopulations

Parameters	*P* value (Blood) *n* = 43	*P* value (Tissue) *n* = 35
Age (≤47 years Vs. > 47 years)	0.755	0.979
Stage (FIGO I Vs. ≥FIGO II)	0.076	0.355
Histology (SCC Vs. ADC)	0.002^#^	0.002^#^
Tumor (≤4 cm Vs. > 4 cm)	0.214	0.032^#^
Stromal invasion (≤1/2 Vs. > 1/2)	0.762	0.555
LN metastasis (negative Vs. positive)	< 0.001^#^	0.001^#^
LVSI (negative Vs. positive)	0.017^#^	0.034^#^
Gran %CD45	0.561	0.957
T %MNC	0.413	0.939
CD4%T	0.066	0.090
CD8%T	0.088	0.005^#^
CD4:CD8	0.048^#^	0.008^#^
rdT %T	0.294	0.317
B %MNC	0.544	0.513
pDC % MNC	0.727	0.278
CD11c + % pDC	0.576	0.509
Baso % MNC	0.528	0.591
BDCA1+ mDC % MNC	0.337	0.132
BDCA3+ mDC % MNC	0.632	0.371
Mono %MNC	0.808	0.812
CD14+ Mo % MNC	0.961	0.481
CD16+ Mo % MNC	0.576	0.062
CD16+ %Mono	0.576	0.062
NK %MNC	0.752	0.533
CD56hi NK % MNC	0.820	0.648
CD56lo NK % MNC	0.525	0.481
CD56hi %NK	0.525	0.563
MDSC % CD33hi	0.576	0.679
DR- %14 + APC	0.443	0.536
DR- %16 + APC	0.093	0.106

*Median value of lymphocyte was used as cut-off value

^#^*P* values with statistical significance were denoted

In addition, we analyzed the correlations of 35 patients’ lymphocyte subsets in the tumor tissue with basic characteristics, and found that some basic characteristics, such as histology (SCC vs. ADC), tumor size (≤4 cm vs. > 4 cm), LN metastasis status (negative vs. positive) and LVSI status (negative vs. positive), exhibited significant difference (*p* < 0.05); however, age (≤47 years vs. > 47 years), stage (FIGO I vs. ≥FIGO II) and stromal invasion (≤1/2 vs. > 1/2) did not (*p* > 0.05) (Table 5). The CD8+ cell proportion and CD4+/CD8+ cell ratio were significantly different (*p* < 0.05), but significant differences were not found for other lymphocyte subsets (*p* > 0.05) (Table 5).

#### CD4+ cells, CD8+ cells and the CD4+/CD8+ cell ratio in the tumor tissue were closely related to recurrence

We followed participants with a median follow-up time of 41 months and compared the distributions of different lymphocyte subsets between recurrent patients and disease-free patients. We found that the distributions of lymphocyte subsets were not significantly different between the recurrent patients and disease-free patients based on analysis of peripheral blood samples from 43 patients. However, in an analysis of tumor tissue samples from 35 patients, the CD4+ cell and CD8+ cell proportions and the CD4+/CD8+ cell ratio were significantly different between the recurrent patients and disease-free patients (*p* < 0.05), but there were no differences in other lymphocyte subsets (Table 6). The results indicated that the distributions of lymphocyte subsets in the peripheral blood may not be the optimal method for detecting recurrence, while the detection of lymphocyte subsets in tumor tissue may be a possible strategy for detecting recurrence.

**Table 6:** Lymphocyte subpopulations: recurrent patients vs. disease-free patients

	Blood (*n* = 43)			Tissue (*n* = 35)		
	Non-recurrent	Recurrent	*P*	Non-recurrent	Recurrent	*P*
Gran %CD45	2.22 ± 4.38	6.65 ± 7.52	0.147	10.66 ± 13.45	8.28 ± 8.24	0.682
T %MNC	57.32 ± 13.27	57.83 ± 11.21	0.921	68.67 ± 19.01	71.23 ± 11.52	0.754
CD4%T	57.45 ± 10.67	48.50 ± 18.24	0.071	48.07 ± 14.41	33.12 ± 13.59	0.018^#^
CD8%T	30.38 ± 8.59	36.41 ± 17.03	0.151	42.87 ± 13.46	57.92 ± 10.76	0.015^#^
CD4:CD8	2.14 ± 0.97	1.65 ± 0.85	0.196	1.32 ± 0.75	0.62 ± 0.34	0.032^#^
rdT %T	7.21 ± 4.54	9.30	0.657	2.73 ± 2.00	2.22 ± 1.23	0.571
B % MNC	6.13 ± 3.31	5.61 ± 1.93	0.693	5.36 ± 3.62	3.65 ± 1.78	0.270
pDC % MNC	0.24 ± 0.12	0.26 ± 0.16	0.696	0.69 ± 0.84	0.47 ± 0.41	0.541
CD11c + % pDC	8.05 ± 5.19	4.63 ± 2.41	0.208	5.92 ± 4.90	5.27 ± 4.20	0.769
Baso % MNC	0.89 ± 0.63	1.36 ± 0.91	0.091	0.10 ± 0.17	0.03 ± 0.08	0.390
BDCA1+ mDC % MNC	0.37 ± 0.19	0.43 ± 0.10	0.595	0.12 ± 0.15	0.07 ± 0.05	0.382
BDCA3+ mDC % MNC	0.02 ± 0.04	0	0.022^#^	0.02 ± 0.07	.0000	0.441
Mono % MNC	14.97 ± 9.08	18.13 ± 10.17	0.391	7.89 ± 7.83	10.98 ± 10.67	0.413
CD14+ Mo % MNC	14.35 ± 7.29	16.63 ± 4.44	0.550	1.90 ± 2.03	3.37 ± 3.23	0.175
CD16+ Mo % MNC	2.91 ± 1.89	4.20 ± 3.91	0.560	5.14 ± 5.30	7.62 ± 7.46	0.357
CD16+ %Mono	17.40 ± 7.24	18.80 ± 12.82	0.745	72.16 ± 13.58	71.30 ± 12.18	0.889
NK %MNC	16.85 ± 9.95	12.37 ± 4.01	0.251	6.12 ± 13.48	3.98 ± 6.32	0.709
CD56hi NK % MNC	0.33 ± 0.15	0.30 ± 0.12	0.693	4.50 ± 12.69	3.40 ± 5.76	0.839
CD56lo NK % MNC	17.68 ± 10.17	14.35 ± 3.59	0.527	1.37 ± 2.10	0.55 ± 0.60	0.357
CD56hi %NK	2.61 ± 2.19	2.05 ± 0.58	0.616	64.10 ± 24.34	74.82 ± 21.91	0.337
MDSC % CD33hi	1.53 ± 2.84	4.08 ± 5.80	0.155	2.67 ± 4.16	1.42 ± 1.30	0.479
DR- %14 + APC	0.58 ± 1.65	0.08 ± 0.15	0.554	16.21 ± 17.23	24.48 ± 22.78	0.335
DR- %16 + APC	1.00 ± 1.68	0.05 ± 0.06	0.272	1.29 ± 2.18	2.75 ± 4.98	0.284

^#^*P* values with statistical significance were denoted

## Discussion

Previous studies have widely noted that the distributions of immunological parameters are changed in patients with cervical cancer and possibly associated with dysfunction in the antitumor immune system [5, 17–20]. The presence of tumor infiltrating lymphocytes that mediate the antitumor immune response has been observed in several types of cancer, including ovarian cancer, breast cancer and pancreatic cancer, and is closely correlated with prognosis [21–23]. However, most previous studies have focused on the distributions of lymphocyte subsets in the peripheral blood, and few studies have revealed the distributions of lymphocyte subpopulations in tumor tissue. Obviously, the lymphocyte subsets in tumor tissue are more representative of the tumor microenvironment than those in the peripheral blood. Therefore, our study evaluated lymphocyte subsets in tumor tissue and peripheral blood samples from cervical cancer patients and precancerous lesion patients. We found inconsistencies in the distributions of lymphocyte subsets between the tumor tissue and peripheral blood samples from the cervical cancer patients and precancerous lesion patients, and the results suggested an imbalance in the homeostasis of the host immune system in the patients with cervical cancer.

The primary result of this study is that total T lymphocyte, CD8+ T cell and granulocyte proportions were significantly higher in the tumor tissue than in the peripheral blood, while CD4+ T cell and rdT cell proportions and the CD4+/CD8+ cell ratio were significantly lower in the tumor tissue than in the peripheral blood from cervical cancer patients. However, when compared with the peripheral blood samples from precancerous lesion patients, those from cervical cancer patients did not exhibit significant differences in lymphocyte subsets. Our results indicated that T cell-mediated immunity was impaired in patients with cervical cancer, which was more distinct in the tumor tissue microenvironment than in the peripheral blood. In addition, our previous study found that a population of highly activated, immunosuppressive HLADR^hi^ Tregs in cervical squamous cell carcinoma and a high frequency of stomal HLADR^hi^ Tregs in patients were significantly associated with relatively poor prognosis [24]. Tregs have fundamental roles in the establishment and maintenance of the peripheral immune tolerance microenvironment [25]. Future research should be conducted to clarify the relationship between HPV infection and Tregs in the tumor tissue and peripheral blood of cervical cancer patients. Increasing granulocyte numbers have been reported to be associated with tumor growth and angiogenesis. A study by Zheng et al. reported that granulocytes but not monocytes significantly promoted tumor growth, implying that granulocytes play key roles in tumor growth and angiogenesis [26]. These findings support the results obtained in our study that the granulocyte proportion was significantly higher in tumor tissue than in the peripheral blood. In addition, a study collected tumor tissue and peripheral blood samples from patients with cervical cancer and found that the CD4+ cell proportion and CD4+/CD8+ cell ratio were significantly lower in cervical cancer tissue than in the peripheral blood, which is consistent with our results [21]. However, the limitation of the previous study was that they had not evaluated lymphocyte subsets other than CD4+ and CD8+ cells; thus, they could not make any interpretations about the correlations of lymphocyte subsets with clinicopathologic parameters or prognostic significance.

Furthermore, our study revealed that the pDCs cell proportion was significantly increased in tumor tissue compared with the peripheral blood, but the BDCA1+ mDCs proportion was significantly decreased. No significant differences were found in CD11c + pDCs or BDCA3+ mDCs between the tumor tissue and the peripheral blood. In addition, total pDC and BDCA1+ mDC proportions in the peripheral blood were significantly lower in cervical cancer patients than in precancerous lesion patients. Dysfunction of DCs induced by tumors can be an important mechanism underlying tumor-induced immune escape because DCs are one of the key types of professional antigen-presenting cells (APCs) that induce tumor-specific immune responses, particularly by cross-priming through MHC class I antigen presentation [27]. Professional APCs are one of the most important inducers of antigen-specific immune responses, and their potentially defective function causes a strong impairment in immunosurveillance in tumor-bearing hosts [28]. The alterations in host lymphocyte subsets that occur in the early stage of tumor progression, including precancerous lesions, may provide some directions to evaluate the roles of different lymphocyte subpopulations in the transition of premalignant lesions into invasive cancer [29].

Our study found that the levels of total monocytes and CD14+ monocytes were significantly decreased in tumor tissue compared to the peripheral blood, while the level of CD16+ monocyte was significantly elevated. The CD16+ monocyte proportion was also significantly increased in the peripheral blood of cervical cancer patients compared to that of precancerous lesion patients. A study by Anna et al. assessed CD14+/CD16+ monocytes in human blood and showed that CD14+/CD16+ monocytes constitute the main subset of blood monocytes involved in the antitumor response and function by producing proinflammatory cytokines and reactive nitrogen and exhibiting increased cytotoxic/cytostatic activity [30]. Therefore, our study revealed that the antitumor response of the host may be defective due to the decreases in total monocyte and CD14+ monocyte levels in tumor tissue.

Our study showed that NK cell and CD56^low^ NK cell proportions were significantly decreased in tumor tissue compared with the peripheral blood, while the percentage of CD56^high^ NK cells was significantly higher in tumor tissue. NK cells are the component of the innate immune surveillance system that plays an important role in host defense immunity. NK cells may be important for the regional immune reaction against cervical carcinoma [31]; thus, the decreases in NK cell subsets in tumor tissue are possibly related to disease progression.

Alterations in the distributions of lymphocyte subsets can occur in the peripheral blood, lymph nodes and other sites in cancer patients, which would induce immune suppression that varies depending on tumor etiology, location, histological type and clinical stage [29]. We detected the associations of lymphocyte subsets with clinicopathological parameters and found that BDCA3+ mDC, total NK cell and CD56^low^ NK cell proportions in the peripheral blood were significantly lower in the tumor size > 4 cm group than in the tumor size ≤4 cm group. In contrast, CD8+ cell, pDC, total monocyte, CD14+ monocyte and CD 16+ monocyte proportions in tumor tissue were significantly higher in the tumor size > 4 cm group than in the tumor size ≤4 cm group. In addition, the CD11c + pDC proportion in the peripheral blood was significantly lower in LVSI-positive patients than in LVSI-negative patients, and LVSI was correlated with a significantly reduced CD8+ cell proportion in tumor tissue. The CD4+ cell proportion and CD4+/CD8+ cell ratio in tumor tissue were significantly reduced. These results indicated some inconsistent implications about clinicopathological parameters between the peripheral blood and tumor tissue. Nevertheless, our results implied that there were close associations between lymphocyte subsets and clinicopathological parameters and that the host immunity system was affected by differences in age, tumor size, LVSI and LN metastasis in cervical cancer patients. Further research should be carried out to clearly reveal the associations between lymphocyte subsets and clinicopathological parameters.

We divided the lymphocyte levels into a low-level group and high-level group by cut-off values defined as the median value for the indicated lymphocyte subset. The results revealed that the CD4+/CD8+ cell ratio was significantly different in both the peripheral blood and tumor tissue when the low level was compared with the high level of the lymphocyte subsets. Mounting evidence has shown that the innate mechanisms that maintain immune cell homeostasis and control the magnitude of immune responses can undergo dramatic dysregulation, which leads to suppression of the immune system and enables tumors to escape immune defense because of the influence of tumor cells [29]. After a median follow-up time of 41 months, we found that the distributions of lymphocyte subsets in the peripheral blood were not significantly different between recurrent patients and disease-free patients. Interestingly, the CD4+ cell proportion and CD4+/CD8+ cell ratio in tumor tissue were significantly decreased in the recurrent patients compared with the disease-free patients, while the CD8+ cell proportion was significantly increased. Our results are similar to those of a previous study by Sheu et al., which reported that a decreased proportion of tumor-infiltrating CD4+ T cells and a reduced CD4+/CD8+ cell ratio were highly correlated with rapid tumor growth and lymph node metastasis due to a poor antitumor response in cervical cancer [31]. A previous study showed that decreased proportions of tumor-infiltrating CD4+ T cells and a reversed CD4+/CD8+ cell ratio were significantly associated with the clinical outcome of patients with cervical cancer [32]. As is well acknowledged, the development of invasive cervical tumors from cervical intraepithelial neoplasia (CIN) is always accompanied by HPV infection [33]. Persistent infection with high-risk HPV in CIN patients may generate an immunosuppressive microenvironment that allows progression to cervical cancer [34]. A direct correlation between HPV infection and the CD4+/CD8+ cell ratio has been reported [35]. A study detected populations of T cells (CD4+ and CD8+ cells) in high-risk HPV-infected precancerous and cancerous lesions of the uterine cervix and found that CD8+ T cell numbers far exceeded CD4+ T cell numbers in invasive cancers [8]. CD4+ and CD8+ T cells have important roles in the natural history of HPV-infected cervical cancer [8].

Our study provides some information about the distributions of lymphocyte subsets in the peripheral blood, tumor tissue and precancerous lesions and reveals the correlations of lymphocyte subsets with clinical characteristics and prognostic variables. Nonetheless, some limitations of our study should be stated, and we should interpret our results with caution. First, some of the analyses could not identify significant differences, which may be due to the lack of a large sample size. We would like to enlarge our sample of participants in future research. In addition, we could not perform subgroup analyses according to different histological types, clinical stages or HPV statuses due to the small sample size. Last, we could not generate survivorship curves according to different distributions of lymphocyte subsets because of the short follow-up time. We will continue to follow our participants and conduct survival analysis in future studies. To provide more knowledge about the distributions of lymphocyte subpopulations in patients with cervical cancer and their relationships with prognosis, a prospective study should be performed.

## Conclusions

Taken together, our results identified significant alterations in lymphocyte subsets in tumor tissue and elucidated that host antitumor immunity may be suppressed. Total T cells, CD4+ cells, CD8+ cells, the CD4+/CD8+ cell ratio, DCs and monocytes in tumor tissue may have significant roles in predicting the homeostasis of the host antitumor immune response. Alterations in lymphocyte subsets were closely correlated with clinicopathologic and prognostic parameters in cervical cancer patients. Further research will lead to a better understanding of the clinical significance of lymphocyte subsets in cervical cancer.

## Supplementary information

**Additional file 1.**

## Data Availability

The dataset supporting the conclusions of this article is available upon request. Please contact Prof. Huijuan Yang (huijuanyang@hotmail.com).

## Abbreviations

Baso: Basophilic Granulocytes
CIN: Cervical Intraepithelial Neoplasia
DCs: Dendritic cells
HIV: Human Immunodeficiency Virus
HPV: Human Papillomavirus
LVSI: Lymph Vascular Space Invasion
mDCs: Myeloid Dendritic Cells
MDSCs: Myeloid-Derived Suppressor Cells
NK: Natural Killer cells
PBMCs: Peripheral Blood Mononuclear Cells
pDCs: Plasmacytoid Dendritic Cells
PFS: Progression-free Survival
