# Supplementary material for: Clinical significance of peripheral blood and tumor tissue lymphocyte subsets in cervical cancer patients
Source: BMC Cancer. 2020 Mar 4;20:173. doi: 10.1186/s12885-020-6633-x (PMC7057584; doi:10.1186/s12885-020-6633-x)
Supplement: Supplementary file 1 — Additional file 1. [file 12885_2020_6633_MOESM1_ESM.doc]

Antibodies and clones against the following proteins were used: CD 1c (L161, BioLegend), CD3 (SK7, BD Biosciences), CD14 (M5E2, BD Biosciences), CD11c (Bu15, eBioscience), CD16 (3G8, BioLegend), CD19 (HIB19, BD Biosciences), CD33 (WM53, BD Biosciences), CD45 (HI30, BioLegend), CD56 (HCD56, BioLegend, or NCAM16, BD Biosciences), CD123 (6H6, eBioscience), BDCA3 (AD5-14H12, Miltenyi), CCR3 (5E8, BioLegend), HLADR (L243, BioLegend), TCRrd (B1.1, eBioscience), TCRVδ1 (REA173, Miltenyi), TCRVδ2 (123R3, Miltenyi) and TCRVa7.2(3C10, BioLegend).
